# Intermediate Filament Protein BFSP2 Controls Spindle Formation via HSC70‐Mediated Stabilization of CLTC During Oocyte meiosis

**DOI:** 10.1002/advs.202506639

**Published:** 2025-07-02

**Authors:** Yu Li, Zihao Zhang, Yu Zhang, Bo Xiong

**Affiliations:** ^1^ College of Animal Sciences Zhejiang University Hangzhou 310058 China; ^2^ College of Animal Science and Technology Nanjing Agricultural University Nanjing 210095 China

**Keywords:** BFSP2, CLTC, HSC70, intermediate filaments, oocyte meiosis, spindle assembly

## Abstract

As a major component of the cytoskeleton, intermediate filaments are generally considered to play a supporting role in mitotic cells. They also take part in the regulation of cell motility, proliferation, differentiation, and apoptosis. However, their specific functions during meiosis are largely unknown. Here, a unique role of an intermediate filament protein beaded filament structural protein 2 (BFSP2) is reported, which is predominantly expressed in lens fiber epithelial cells, as a spindle formation controller in oocyte meiosis. BFSP2 is constantly expressed during oocyte meiotic maturation, and specifically distributed on the spindle apparatus at metaphase I (MI) and metaphase II (MII) stages. Depletion of BFSP2 resulted in the meiotic arrest at MI stage due to the aberrant spindle assembly‐induced spindle assembly checkpoint activation. Depletion of BFSP2 also led to incorrect kinetochore‐microtubule attachments and the occurrence of aneuploidy in oocytes. Mechanistically, immunoprecipitation combined with mass spectrometry analysis identified clathrin heavy chain 1 (CLTC) as the downstream mediator of BFSP2 during meiotic spindle assembly. It is further determined that BFSP2 recruited the molecular chaperone heat shock cognate protein 70 (HSC70) to the spindle apparatus for stabilizing CLTC, and thus driving the spindle formation. In summary, these findings uncover a noncanonical function of the intermediate filament protein BFSP2 as a spindle assembly controller in oocyte meiosis.

## Graphical abstract

Meiosis is a specialized form of cell division that has different regulation and mechanisms with mitosis in numerous aspects. Particularly, meiosis I is unique and occurs only in germ cells to separate homologous chromosomes. Thus, determining how this unusual chromosome segregation behavior is established is central to understanding germ cell development. The current study uncovers that an intermediate filament protein BFSP2 exerts a previously unknown function in the spindle assembly to promote the meiotic progression and maintain the chromosome euploidy in oocyte meiosis.

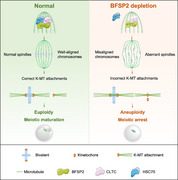

## 1 Introduction

Microfilaments (MFs), microtubules (MTs), and intermediate filaments (IFs) together constitute the fiber network system of the cytoskeleton in eukaryotic cells.^[^ ^1^, ^2^, ^3^, ^4^ ^]^ Although these three types of fibers have many differences in morphology, structure, and function, they are interconnected to participate in a wide of cellular processes such as cell shape support, cell division, intracellular transport, cell mobility, signal transduction, and organelle positioning.^[^ ^5^, ^6^, ^7^, ^8^, ^9^, ^10^ ^]^ Among these three fibers, the size of IFs is intermediate in diameter, typically ranging from 8 to 12 nm, which is between the MFs (≈6 nm) and MTs (≈25 nm).^[^ ^11^, ^12^, ^13^, ^14^ ^]^ IFs are composed of a variety of protein subunits, depending on the cell types and tissues. At least 50 different IF proteins have been identified and classified into different groups based on similarities between their amino acid sequences, including keratins (epithelial cells), vimentin (mesenchymal cells), neurofilaments (neurons), desmin (muscle cells), and lamins (nuclear envelope).^[^ ^15^, ^16^ ^]^

Interestingly, beaded filament structural protein 2 (BFSP2) is an orphan IF protein because it does not belong to any of the well‐characterized IF family categories.^[^ ^17^ ^]^ BFSP2 is abundantly expressed in the differentiated fiber cells of the lens and plays a critical role in maintaining the morphology of lens cells, facilitating their motility, and ensuring lens transparency.^[^ ^18^, ^19^, ^20^, ^21^ ^]^ Previous reports have shown that knockout of *Bfsp2* in mice induces significant alterations in the morphology of the IF cytoskeleton in lens fiber cells and loss of lens optical properties.^[^ ^22^, ^23^, ^24^ ^]^ In humans, deletion or mutation of the *BFSP2* gene has been linked to the development of hereditary cataracts.^[^ ^18^, ^25^, ^26^, ^27^ ^]^

Oocyte meiosis is a unique form of cell division that must undergo two rounds of meiotic division to produce haploid gametes with the correct chromosomal number.^[^ ^28^, ^29^ ^]^ However, for most mammalian species, particularly in humans, oocyte meiosis is prone to errors. Oocytes with incorrect chromosome numbers can give rise to aneuploid embryos after fertilization, which is a leading cause of pregnancy loss and developmental disorders.^[^ ^30^ ^]^ At the core of chromosome segregation is the spindle, whose primary function is to organize and coordinate the distribution of chromosomes during cell division. Accurate chromosome segregation during the meiotic maturation of the oocyte relies heavily on the formation of a well‐organized bipolar spindle apparatus and the proper interactions between chromosomes and microtubules.^[^ ^31^ ^]^ Therefore, well‐coordinated spindle dynamics and precise chromosome segregation are crucial determinants of oocyte quality.^[^ ^32^ ^]^

In the present study, we discovered that BFSP2 was also expressed in oocytes during meiotic maturation, and specifically accumulated on the spindle apparatus at metaphase I and metaphase II stages. Loss of function experiments indicated that BFSP2 is required for the spindle assembly, meiotic progression, and chromosome euploidy maintenance in oocytes. We further elucidated the molecular mechanism of BFSP2 in regulating spindle assembly, and evidenced that its function is conserved between mouse and porcine oocytes.

## 2 Results

### 2.1 Protein Expression and Subcellular Localization of BFSP2 During Mouse Oocyte Maturation

**Figure 1:**
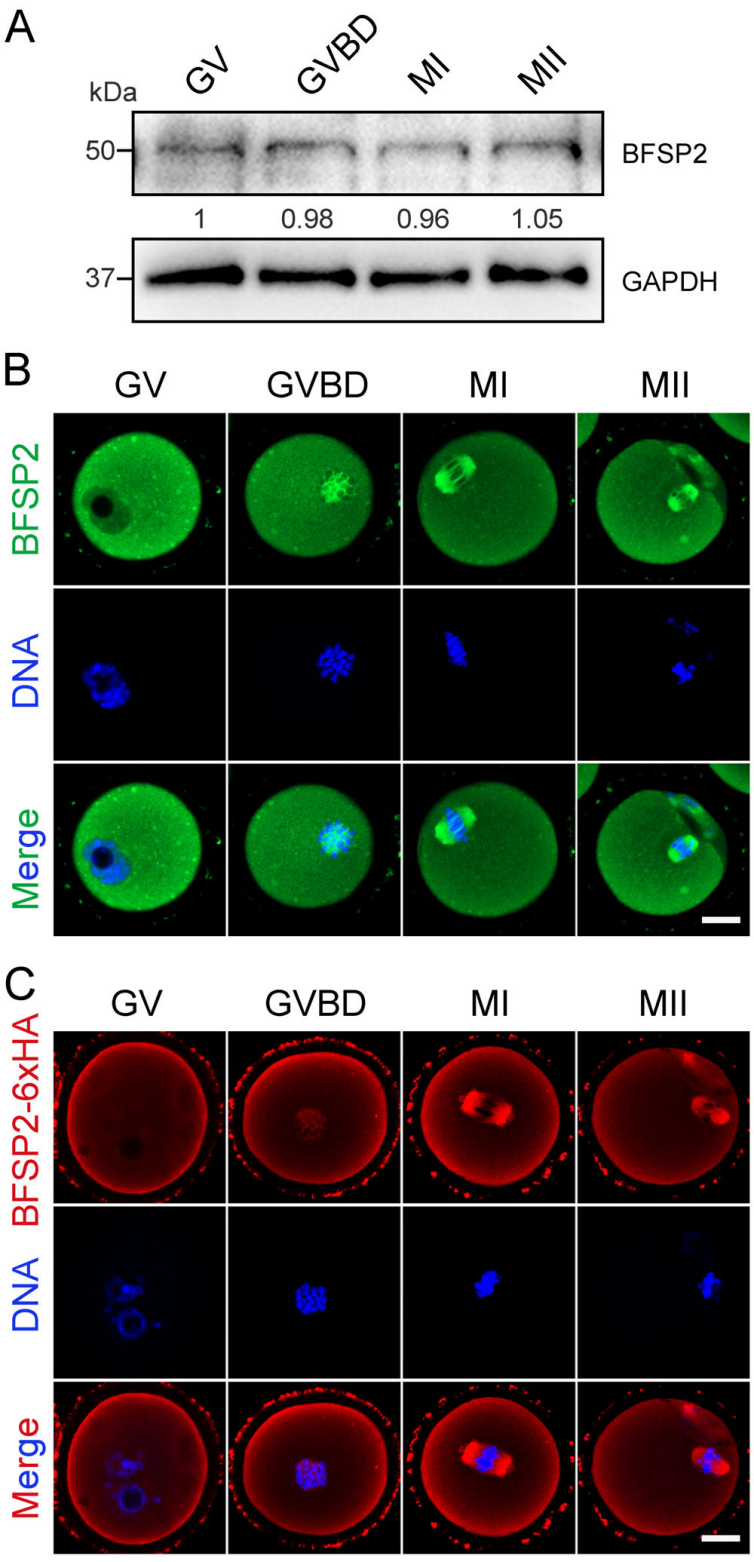
Protein expression and subcellular localization of BFSP2 during mouse oocyte meiosis. A) Protein levels of BFSP2 in oocytes at different developmental stages corresponding to GV, GVBD, MI, and MII were examined by immunoblotting analysis. The blots were probed with BFSP2 and GAPDH antibodies, respectively. B) Fluorescence images of BFSP2 localization in oocytes. Mouse oocytes at GV, GVBD, MI and MII stages were immunostained with BFSP2 antibody and counterstained with Hoechstl. Scale bar, 20 µm. C) Fluorescence images of BFSP2‐6×HA localization in oocytes. Mouse oocytes at GV, GVBD, MI, and MII stages were immunostained with HA antibody and counterstained with Hoechst. Scale bar, 20 µm.

We first of all examined the protein expression of BFSP2 during oocyte meiotic maturation. As assessed by immunoblotting, BFSP2 was constantly expressed at all maturational stages in oocytes, including germinal vesicle (GV), germinal vesicle breakdown (GVBD), metaphase I (MI), and metaphase II (MII) stages (**Figure** **1**A). Immunostaining data further verified the expression of endogenous BFSP2 in oocytes, and showed that BFSP2 distributed throughout the cytoplasm, but was enriched on the spindle apparatus at MI and MII stages (Figure 1B). We also investigated the subcellular localization of vimentin, another well‐characterized IF protein, and did not observe its spindle‐like accumulation in oocytes (Figure S1, Supporting Information), indicating that the spindle localization pattern is unique to BFSP2.

In addition, we overexpressed BFSP2‐6×HA mRNA in oocytes to observe the expression and localization of exogenous BFSP2. Immunoblotting results validated the protein expression of BFSP2‐6×HA in oocytes (Figure S2, Supporting Information). Consistent with the localization pattern of endogenous BFSP2, BFSP2‐6×HA also accumulated on the spindle apparatus as assessed by immunostaining analysis (Figure 1C). Collectively, these observations suggest that BFSP2 might play a role in the spindle dynamics during oocyte meiosis.

### 2.2 Depletion of BFSP2 Leads to the Meiotic Arrest by Activating SAC in Oocytes

**Figure 2:**
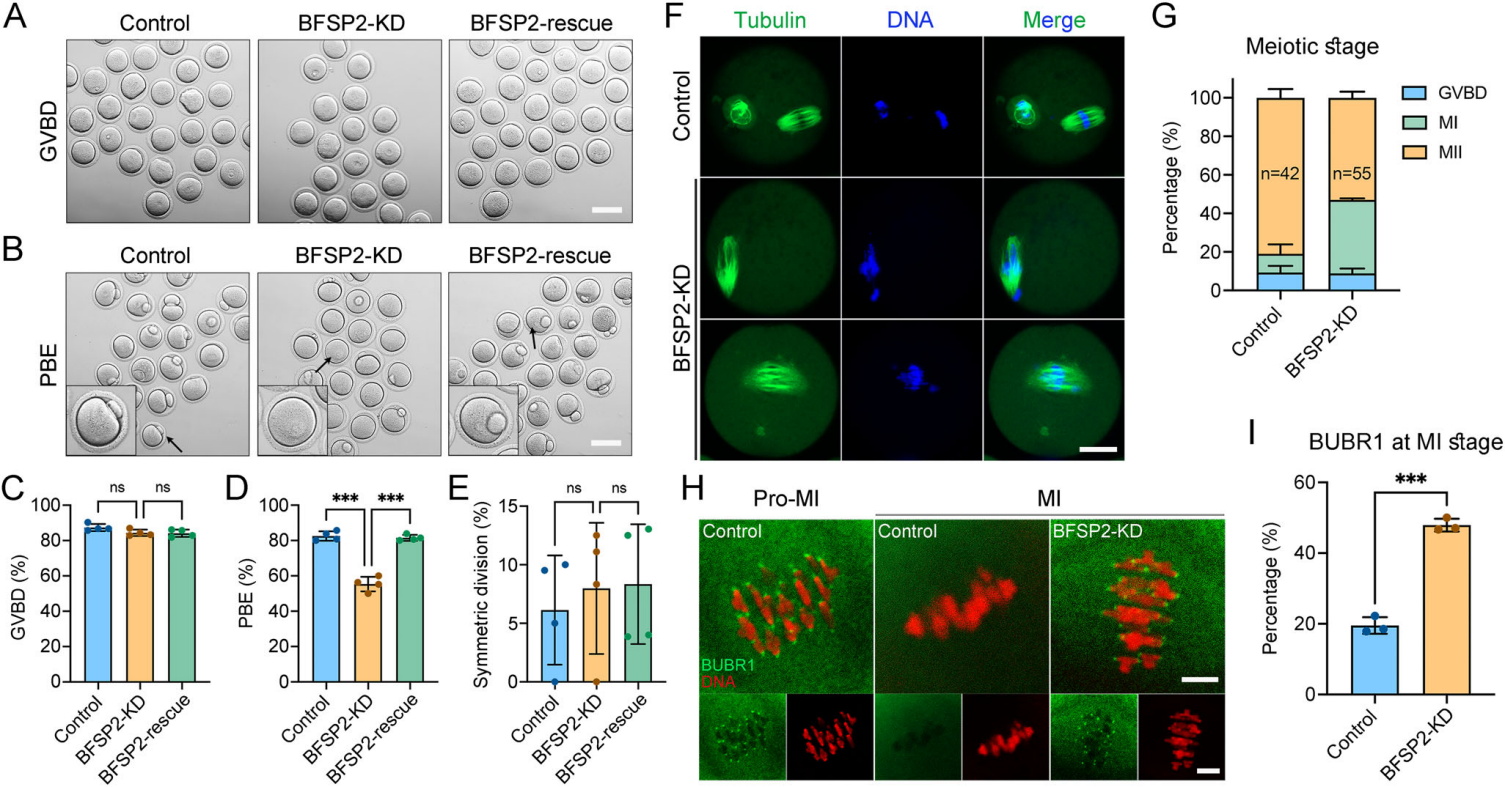
Effects of BFSP2 depletion on the mouse oocyte meiotic progression and SAC activation. (A) Representative images of oocytes at GVBD stage in control, BFSP2‐KD, and BFSP2‐rescued (BFSP2‐KD + BFSP2‐6×HA) groups. Scale bar, 80 µm. B) Representative images of oocytes after in vitro maturation in control, BFSP2‐KD, and BFSP2‐rescued groups. Scale bar, 80 µm. C) The GVBD rate was quantified in control (*n* = 119), BFSP2‐KD (n = 120), and BFSP2‐rescued (*n* = 141) oocytes. D) The PBE rate was quantified in control (*n* = 119), BFSP2‐KD (*n* = 120), and BFSP2‐rescued (*n* = 141) oocytes. E) The rate of symmetric division was quantified in control (*n* = 119), BFSP2‐KD (*n* = 120), and BFSP2‐rescued (*n* = 141) oocytes. F) Fluorescence images of the spindle and chromosome in control and BFSP2‐KD oocytes after in vitro maturation. Oocytes were stained with α‐tubulin to show the spindle and counterstained with Hoechst to visualize the chromosome. Scale bar, 20 µm. G) The proportion of oocytes at GVBD, MI, and MII stages in control (*n* = 42) and BFSP2‐KD (*n* = 55) groups after in vitro maturation. H) Fluorescence images of BUBR1 on the chromosomes in control and BFSP2‐KD oocytes at pro‐metaphase I (pro‐MI) and MI stages. Oocytes were immunostained with BUBR1 antibody and counterstained with PI. Scale bars, 5 µm. I) The proportion of BUBR1 presence on the chromosomes at MI stage was quantified in control (*n* = 82) and BFSP2‐KD (*n* = 48) oocytes. Data in (C–E), G), and I) were expressed as mean ± SEM of at least three independent experiments. ^***^ *P* < 0.001; ns, no significance.

We next performed loss‐of‐function experiments using BFSP2‐targeting siRNAs. The knockdown efficiency of BFSP2 was assessed by immunoblotting analysis, showing a more than 70% reduction of BFSP2 protein levels in BFSP2‐depleted oocytes (Figure S3, Supporting Information). Following BFSP2 depletion, we traced the oocyte meiotic progression by observing the occurrence of GVBD and polar body extrusion (PBE), and found that BFSP2 depletion impaired the PBE instead of GVBD (**Figure** **2**A–D), suggesting that BFSP2 is essential for the completion of oocyte meiosis I. Meanwhile, BFSP2 depletion did not produce the big polar body, indicating that BFSP2 is dispensable for the asymmetric division of oocytes (Figure 2B,E). To exclude the possibility that these meiotic defects might be caused by the off‐target effect of siRNA, we expressed exogenous BFSP2‐6×HA in BFSP2‐depleted oocytes to restore the BFSP2 function. As anticipated, expression of BFSP2‐6×HA significantly increased the incidence of PBE in BFSP2‐depleted oocytes (Figure 2B,D), which corroborates the function of BFSP2 during oocyte meiotic maturation.

To further determine which stage BFSP2‐depleted oocytes arrest during meiotic progression, we stained the spindle and chromosome for evaluation. As shown in Figure 2F,G, quantification of oocytes at different stages revealed that those oocytes that did not reach the MII stage following BFSP2 depletion were predominantly arrested at MI stage (Figure 2F,G). Given that persistent activation of the spindle assembly checkpoint (SAC) is the main cause leading to the MI arrest, we then stained the SAC component BUBR1 in control and BFSP2‐depleted oocytes. The immunostaining results manifested that BUBR1 signals were present on the chromosomes at pro‐MI stage but vanished at MI stage in control oocytes (Figure 2H,I). Whereas, BUBR1 signals still remained on the chromosomes at MI stage in BFSP2‐depleted oocytes (Figure 2H,I), implicating that loss of BFSP2 caused the oocyte meiotic arrest by inducing SAC activation.

### 2.3 Depletion of BFSP2 Results in Spindle/Chromosome Defects and Aneuploidy in Oocytes

**Figure 3:**
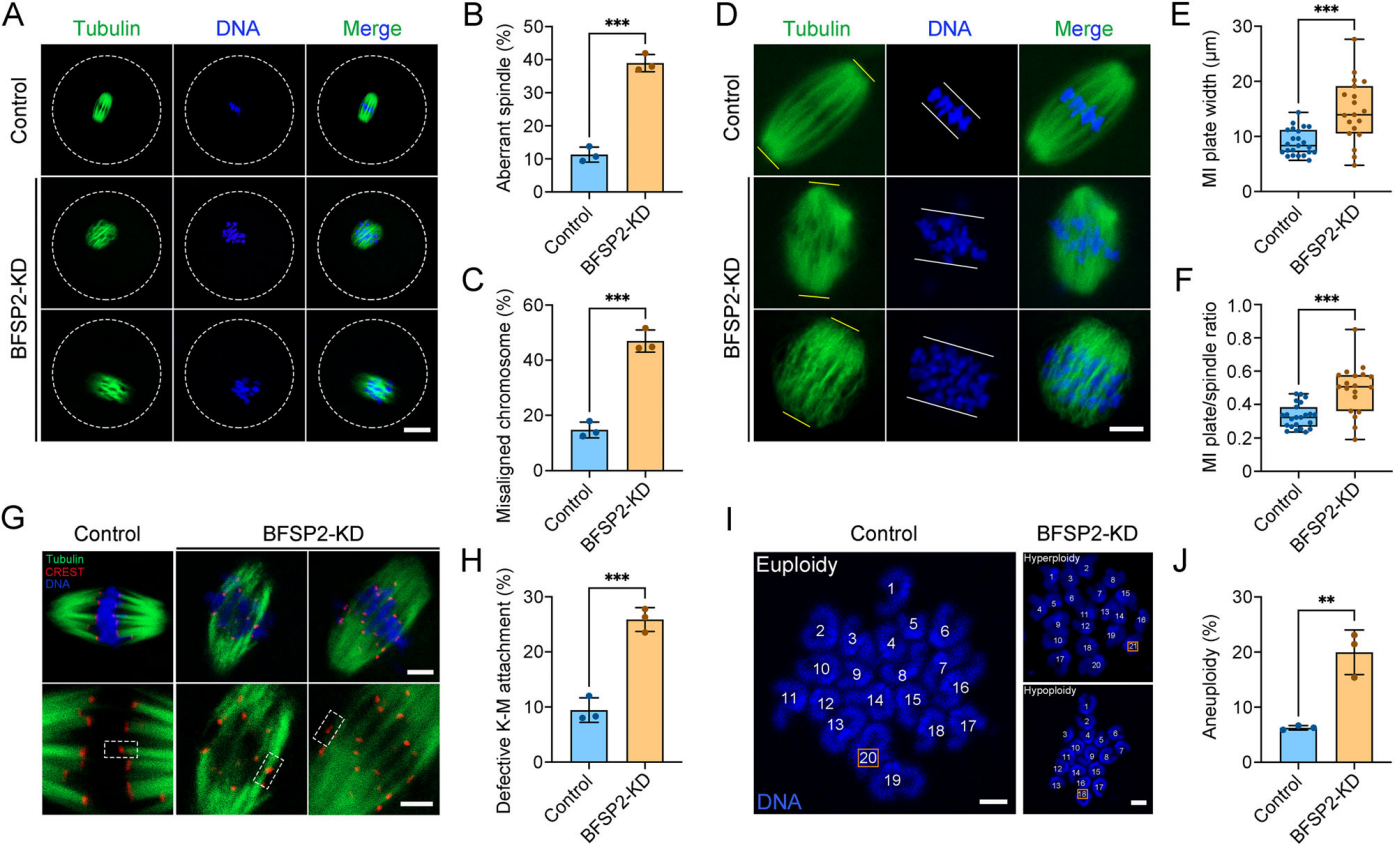
Effects of BFSP2 depletion on the spindle/chromosome structure, K‐M attachments, and chromosome euploidy in mouse oocytes. A) Fluorescence images of spindle morphologies and chromosome alignment in control and BFSP2‐KD oocytes at MI stage. Oocytes were stained with α‐tubulin to show the spindle and counterstained with Hoechst to visualize the chromosome. Scale bar, 20 µm. B) The rate of abnormal spindles was quantified in control (*n* = 89) and BFSP2‐KD (*n* = 87) oocytes. C) The rate of misaligned chromosomes was quantified in control (*n* = 89) and BFSP2‐KD (*n* = 87) oocytes. D) Fluorescence images of spindle length and MI plate width in control and BFSP2‐KD oocytes. Oocytes were stained with α‐tubulin to show the spindle and counterstained with Hoechst to visualize the chromosome. Yellow lines indicate the spindle length, and white lines represent the MI plate width. Scale bar, 10 µm. E) The width of MI plate was measured in control (*n* = 23) and BFSP2‐KD (*n* = 19) oocytes. F) The MI plate width/spindle length ratio was quantified in control (*n* = 23) and BFSP2‐KD (*n* = 19) oocytes. G) Fluorescence images of K‐M attachments in control and BFSP2‐KD oocytes at MI stage. Oocytes cultured to MI stage were incubated at 4 °C for 5 min for cold treatment to depolymerize unstable microtubules, and then stained with α‐tubulin to show the spindle, stained with CREST to display the kinetochore, and counterstained with Hoechst to visualize the chromosome. Scale bars, 5 µm. H) The rate of defective K‐M attachments was quantified in control (*n* = 74) and BFSP2‐KD (*n* = 55) oocytes. I) Fluorescence images of euploid and aneuploid oocytes in control and BFSP2‐KD groups. Chromosome spreading was performed to count the number of chromosomes stained with Hoechst in control and BFSP2‐KD oocytes at MII stage. Scale bar, 2 µm. J) The rate of aneuploidy was quantified in control (*n* = 48) and BFSP2‐KD (*n* = 40) oocytes. Data in (B), (C), (H), and (J) were expressed as mean ± SEM, and (E,F) were expressed as mean ± SD of at least three independent experiments. ^**^ *P* < 0.01; ^***^ *P* < 0.001.

Considering the spindle‐like localization pattern of BFSP2, as well as its role in activation of SAC, we speculated that BFSP2 may be involved in the spindle dynamics. We then stained the oocytes with tubulin antibody and Hoechst to visualize the spindle and chromosome structure. Fluorescence imaging and quantification data demonstrated that normally organized spindles with well‐aligned chromosomes at the equatorial plate were observed in most control oocytes (**Figure** **3**A–C). In contrast, BFSP2 depletion dramatically elevated the frequency of various morphology‐aberrant spindles and misaligned chromosomes (Figure 3A–C). In addition, the chromosome abnormalities in BFSP2‐depleted oocytes were further confirmed by the measurement of width of MI plate and ratio of MI plate width to spindle length, showing the considerably increased MI plate (Figure 3D–F).

The spindle/chromosome abnormalities may be related to the incorrect kinetochore‐microtubule (K‐M) attachments. To test it, we applied a cold treatment on oocytes to depolymerize microtubules that were not correctly attached to kinetochores. In the control oocytes, we observed that microtubule fibers captured kinetochores on the well‐aligned chromosomes (Figure 3G). While in BFSP2‐depleted oocytes, many kinetochores were not attached by microtubule fibers, or improperly attached (Figure 3G). Quantitative analysis showed a significant increase in the proportion of defective K‐M attachments in BFSP2‐depleted oocytes compared to the controls (Figure 3H).

These K‐M attachment errors will inevitably lead to the chromosome missegregation and aneuploidy.^[^ ^31^, ^33^, ^34^, ^35^ ^]^ Hence, we performed chromosome spreading to test it. The staining results showed that most oocytes contained 20 univalents in the control group (Figure 3I). However, more than or less than 20 univalents were observed in BFSP2‐depleted oocytes (Figure 3I). Quantitatively, the aneuploidy rate was significantly increased in BFSP2‐depleted oocytes in comparison with the controls (Figure 3J). Altogether, our data implies that BFSP2 is indispensable for proper spindle/chromosome structure and K‐M attachments to maintain the euploidy in oocytes.

### 2.4 BFSP2 Interacts with CLTC and Affects its Protein Stability in Oocytes

**Figure 4:**
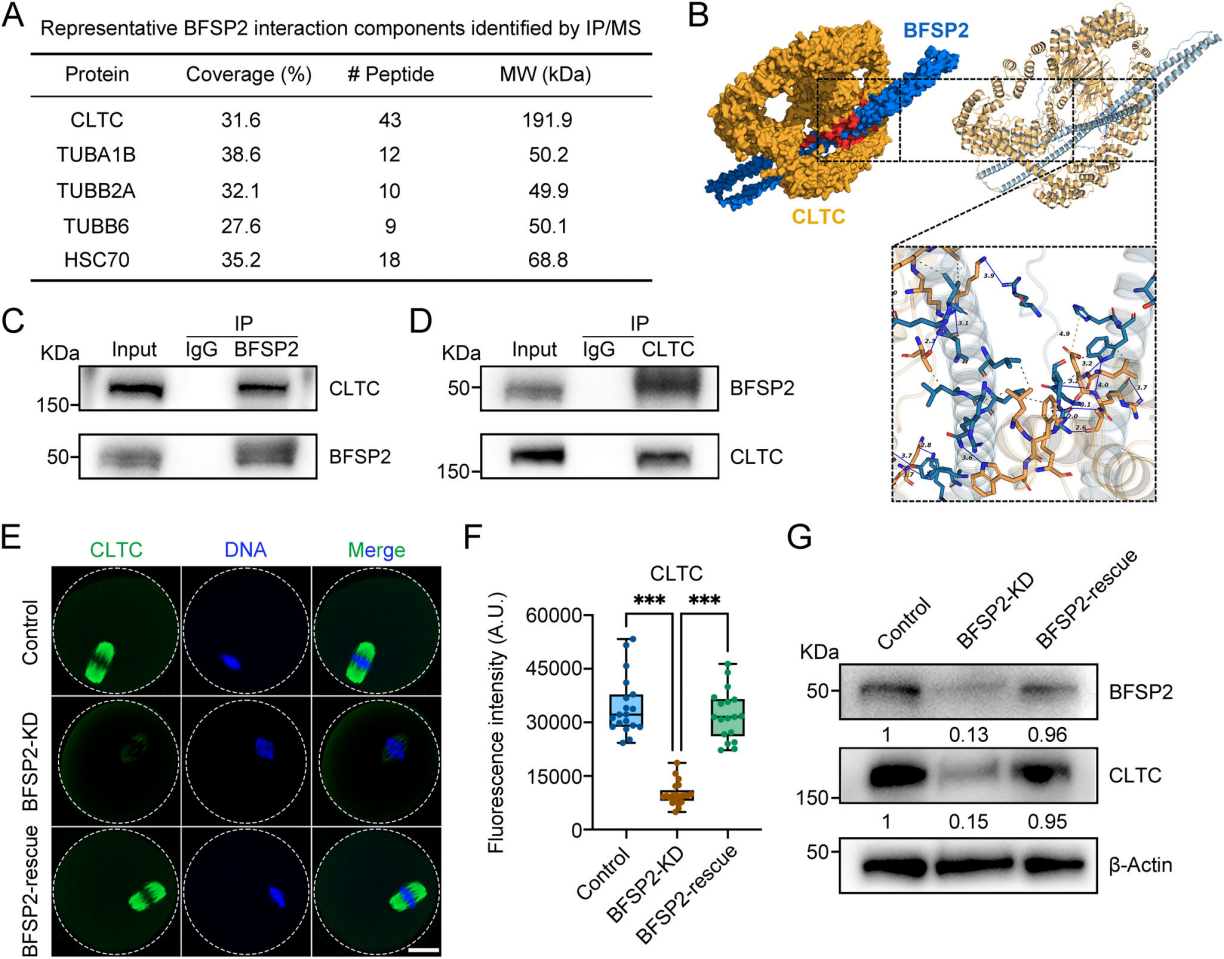
Interaction between BFSP2 and CLTC. A) Representative binding proteins of BFSP2 identified by IP/MS analysis. Protein name, coverage percentage, number of peptides, and molecular weight (MW) were shown in the table. B) Representation of the 3D structure and predicted interaction of mouse BFSP2 and CLTC using AlphaFold databank by HDOCK server. C) Co‐IP using BFSP2 antibody followed by immunoblotting analysis with CLTC and BFSP2 antibodies. D) Co‐IP using CLTC antibody followed by immunoblotting analysis with BFSP2 and CLTC antibodies. E) Fluorescence images of CLTC in control, BFSP2‐KD, and BFSP2‐rescued oocytes at MI stage. Scale bar, 20 µm. F) The fluorescence intensity of CLTC signals was quantified in control (*n* = 19), BFSP2‐KD (*n* = 21), and BFSP2‐rescued (*n* = 18) oocytes. G) Protein levels of CLTC in control, BFSP2‐KD, and BFSP2‐rescued oocytes as assessed by immunoblotting analysis. The blots were probed with BFSP2, CLTC, and β‐Actin antibodies, respectively. Data in (F) were expressed as mean ± SD of at least three independent experiments. ^**^ *P* < 0.01; ^***^ *P* < 0.001.

To elucidate the molecular mechanisms underlying the meiotic defects in oocytes depleted of BFSP2, we performed immunoprecipitation/mass spectrometry (IP/MS) assay using BFSP2 antibody to identify the proteins that interact with BFSP2. Among the top candidates in the list of MS data, we noticed one molecule, clathrin heavy chain 1 (CLTC) (**Figure** **4**A), because it has been implicated in the spindle assembly in mammalian oocytes.^[^ ^36^, ^37^, ^38^ ^]^ We then applied AlphaFold database to predict the 3D protein structures of BFSP2 and CLTC,^[^ ^39^, ^40^ ^]^ and docked these two proteins by HDOCK server to simulate their interaction.^[^ ^41^, ^42^, ^43^, ^44^, ^45^ ^]^ The score of docking model was ‐276.37, with 0.93 of confidence score, indicative of a high possibility of interaction between BFSP2 and CLTC (Figure 4B). In addition, we verified this interaction by conducting co‐immunoprecipitation (co‐IP) experiments. Immunoblotting analysis revealed that CLTC was present in the BFSP2 precipitate, and vice versa, BFSP2 was observed in CLTC precipitate, confirming the binding of CLTC to BFSP2 (Figure 4C,D). Moreover, we also found that both endogenous and exogenous CLTC exhibited a spindle‐like localization pattern in oocytes, similar to BFSP2 (Figure S4, Supporting Information).

To further clarify how BFSP2 impacts CLTC dynamics, we examined the distribution and protein expression of CLTC in BFSP2‐depleted oocytes. Immunostaining results indicated that BFSP2 depletion did not affects the normal spindle‐like distribution of CLTC in oocytes, but substantially decreased its abundance, particularly in the spindle region as assessed by fluorescence intensity quantification, which can be recovered by expression of BFSP2‐6×HA (Figure 4E,F). Consistently, immunoblotting data validated that the total protein levels of CLTC were lowered in BFSP2‐depleted oocytes, but restored by expression of exogenous BFSP2 (Figure 4G), suggesting that the protein homeostasis of CLTC requires BFSP2 in oocytes.

### 2.5 BFSP2 Exerts its Function though CLTC in Oocytes

**Figure 5:**
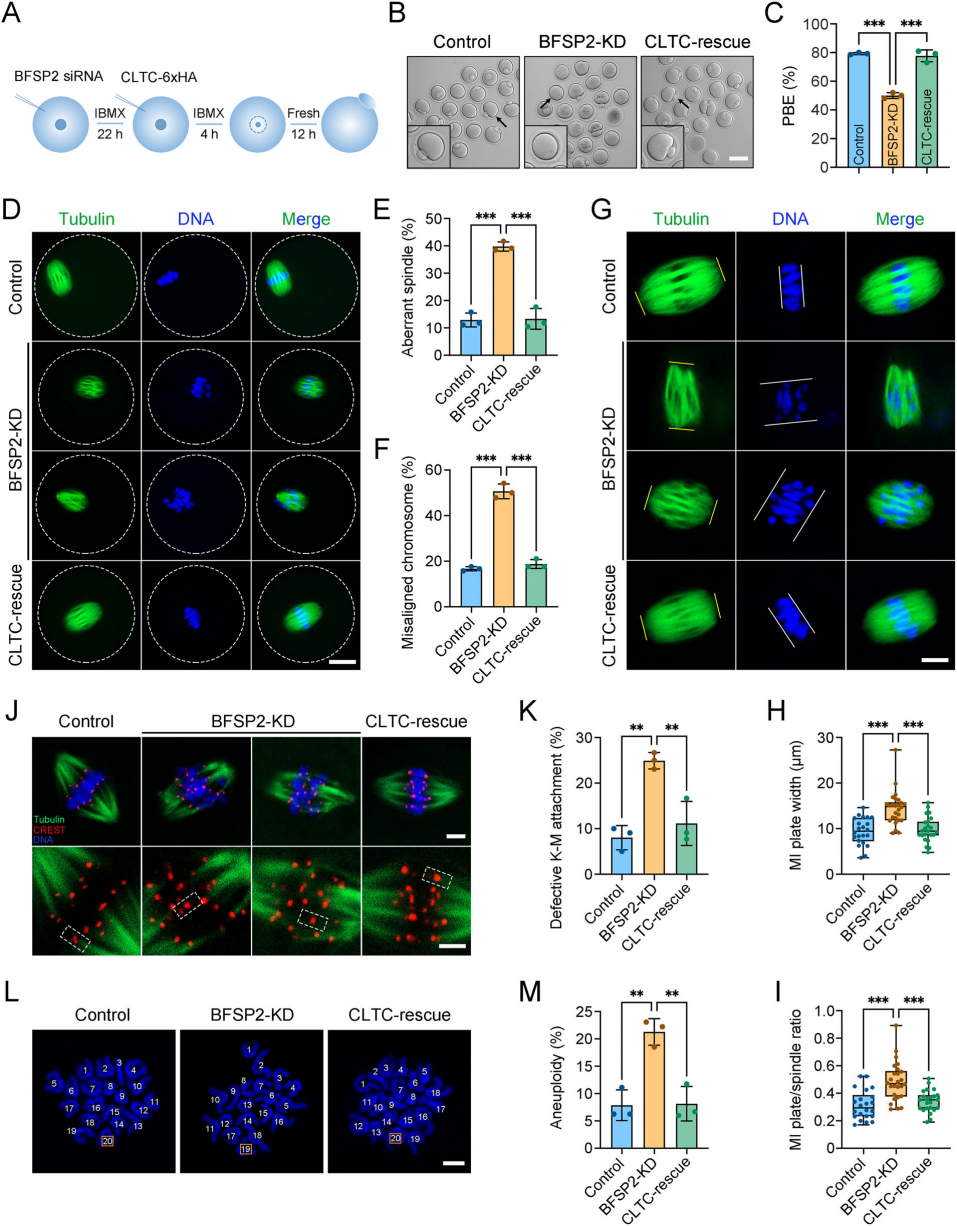
Expression of CLTC‐6×HA in BFSP2‐depleted oocytes alleviates the meiotic defects. A) Schematic flowchart of the expression of CLTC‐6×HA in BFSP2‐depleted oocytes. B) Representative images of oocytes after in vitro maturation in control, BFSP2‐KD, and CLTC‐rescued groups. Scale bar, 80 µm. C) The PBE rate was quantified in control (*n* = 91), BFSP2‐KD (*n* = 89), and CLTC‐rescued (*n* = 88) oocytes. (D) Fluorescence images of spindle morphologies and chromosome alignment in control, BFSP2‐KD, and CLTC‐rescued oocytes at MI stage. Oocytes were stained with α‐tubulin to show the spindle and counterstained with Hoechst to visualize the chromosome. Scale bar, 20 µm. E) The rate of abnormal spindles was quantified in control (*n* = 54), BFSP2‐KD (*n* = 73), and CLTC‐rescued (*n* = 53) oocytes. F) The rate of misaligned chromosomes was quantified in control (*n* = 54), BFSP2‐KD (*n* = 73), and CLTC‐rescued (*n* = 53) oocytes. G) Fluorescence images of spindle length and MI plate width in control, BFSP2‐KD, and CLTC‐rescued oocytes. Oocytes were stained with α‐tubulin to show the spindle and counterstained with Hoechst to visualize the chromosome. Yellow lines indicate the spindle length, and white lines represent the MI plate width. Scale bar, 10 µm. H) The width of MI plate was measured in control (*n* = 22), BFSP2‐KD (*n* = 26), and CLTC‐rescued (*n* = 23) oocytes. I) The MI plate width/spindle length ratio was quantified in control (*n* = 22), BFSP2‐KD (*n* = 26), and CLTC‐rescued (*n* = 23) oocytes. J) Fluorescence images of K‐M attachments in control, BFSP2‐KD, and CLTC‐rescued oocytes at MI stage. Oocytes cultured to MI stage were incubated at 4 °C for 5 min for cold treatment to depolymerize unstable microtubules, and then stained with α‐tubulin to show the spindle, stained with CREST to display the kinetochore, and counterstained with Hoechst to visualize the chromosome. Scale bars, 5 µm. K) The rate of defective K‐M attachments was quantified in control (*n* = 62), BFSP2‐KD (*n* = 44), and CLTC‐rescued (*n* = 36) oocytes. L) Fluorescence images of euploid and aneuploid oocytes in control, BFSP2‐KD, and CLTC‐rescued groups. Chromosome spreading was performed to count the number of chromosomes stained with Hoechst in control, BFSP2‐KD, and CLTC‐rescued oocytes at MII stage. Scale bar, 2 µm. M) The rate of aneuploidy was quantified in control (*n* = 50), BFSP2‐KD (*n* = 80), and CLTC‐rescued (*n* = 49) oocytes. Data in (C), (E), (F), (K), and (M) were expressed as mean ± SEM, and (H,I) were expressed as mean ± SD of at least three independent experiments. ^**^ *P* < 0.01; ^***^ *P* < 0.001.

To determine whether CLTC is the downstream molecule mediating the function of BFSP2, we then tested the possibility that restoration of CLTC levels can mitigate the meiotic failure in BFSP2‐depleted oocytes by expression of exogenous CLTC‐6×HA (**Figure** **5**A). Immunoblotting results displayed that CLTC‐6×HA was successfully expressed in oocytes (Figure S5A, Supporting Information), and CLTC protein levels were restored in CLTC‐rescued oocytes (Figure S5B, Supporting Information). Consistent with the above observations, the PBE rate was remarkably reduced in BFSP2‐depleted oocytes compared to the controls, but increased after expression of exogenous CLTC in CLTC‐rescued oocytes (Figure 5B,C). Additionally, expression of CLTC‐6×HA also decreased the proportions of spindle/chromosome abnormalities, K‐M attachment defects, and aneuploidy occurring in BFSP2‐depleted oocytes (Figure 5D–M), demonstrating that the specific role of BFSP2 in oocyte meiosis is mediated by CLTC.

### 2.6 BFSP2 Recruits HSC70 to Stabilize CLTC in Oocytes

**Figure 6:**
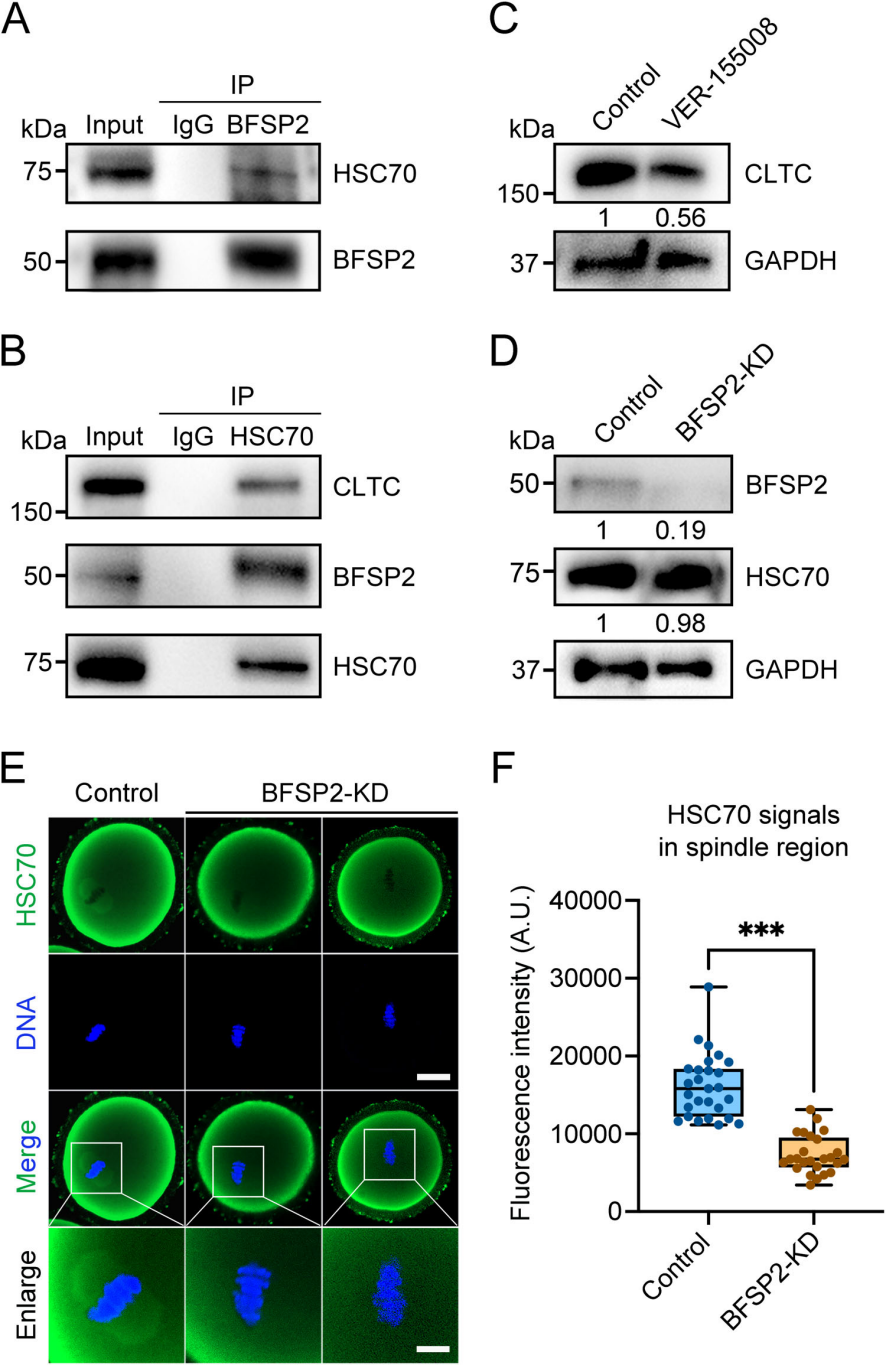
BFSP2 recruits HSC70 to stabilize CLTC. A) Co‐IP using BFSP2 antibody followed by immunoblotting analysis with HSC70 and BFSP2 antibodies. B) Co‐IP using HSC70 antibody followed by immunoblotting analysis with CLTC, BFSP2, and HSC70 antibodies. C) Protein levels of CLTC in control and VER‐155008‐treated oocytes as assessed by immunoblotting analysis. The blots were probed with CLTC and GAPDH antibodies, respectively. D) Protein levels of HSC70 in control and BFSP2‐KD oocytes as assessed by immunoblotting analysis. The blots were probed with BFSP2, HSC70, and GAPDH antibodies, respectively. E) Fluorescence images of HSC70 localization in control and BFSP2‐KD oocytes at MI stage. Oocytes were immunostained with HSC70 antibody and counterstained with Hoechst. Scale bars, 20, 10 µm. F) The fluorescence intensity of HSC70 signals in the spindle region was quantified in control (*n* = 27) and BFSP2‐KD (*n* = 25) oocytes. Data in (F) were expressed as mean ± SD of at least three independent experiments. ^***^ *P* < 0.001.

We next explored the potential mechanism by which BFSP2 regulates CLTC protein levels in oocytes. When we reviewed the MS data, we found that a molecular chaperone protein heat shock cognate protein 70 (HSC70), which has been reported to take part in microtubule dynamics,^[^ ^46^ ^]^ is also a potential binding partner of BFSP2. Immunostaining results showed that HSC70 exhibited a spindle‐like localization in oocytes, similar to BFSP2 and CLTC (Figure S6, Supporting Information). Co‐IP experiments validated the interactions between BFSP2 and HSC70, as well as HSC70 and CLTC (**Figure** **6**A,B). In addition, inhibition of HSC70 with its potent inhibitor VER‐155008 lowered CLTC protein levels in oocytes (Figure 6C), indicating that HSC70 is necessary for CLTC stability. We further revealed that BFSP2 depletion had no effect on the total protein levels of HSC70 in oocytes as evaluated by immunoblotting (Figure 6D), but decreased the abundance of HSC70 on the spindle region as displayed by immunofluorescence (Figure 6E,F). Therefore, these findings indicate that BFSP2 is crucial for recruiting HSC70 to the spindle apparatus, thereby stabilizing CLTC.

### 2.7 The Meiotic Function of BFSP2 in Oocytes is Conserved between Mouse and Pig

**Figure 7:**
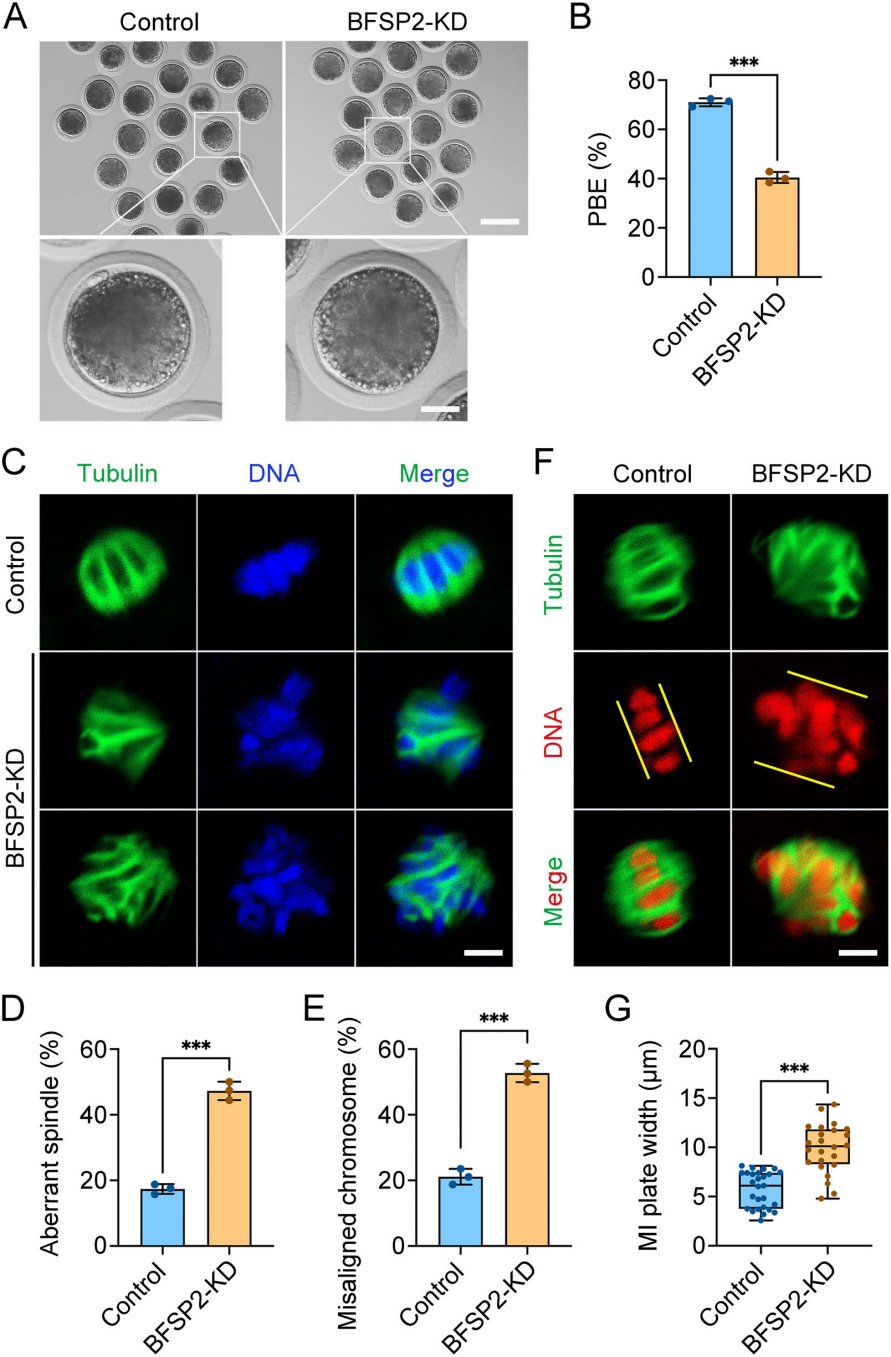
Effects of BFSP2 depletion on the porcine oocyte meiosis. A) Representative images of porcine oocytes after in vitro maturation in control and BFSP2‐KD groups. Scale bars, 120, 30 µm. B) The PBE rate was quantified in control (*n* = 83) and BFSP2‐KD (*n* = 79) oocytes. C) Fluorescence images of spindle morphologies and chromosome alignment in control and BFSP2‐KD oocytes at MI stage. Oocytes were stained with α‐tubulin to show the spindle and counterstained with Hoechst to visualize the chromosome. Scale bar, 5 µm. D) The rate of abnormal spindles was quantified in control (*n* = 52) and BFSP2‐KD (*n* = 55) oocytes.E) The rate of misaligned chromosomes was quantified in control (*n* = 52) and BFSP2‐KD (*n* = 55) oocytes. F) Fluorescence images of MI plate width in control and BFSP2‐KD oocytes. Oocytes were stained with α‐tubulin to show the spindle and counterstained with PI to visualize the chromosome. Yellow lines indicate the MI plate width. Scale bar, 5 µm. G) The width of MI plate was measured in control (*n* = 27) and BFSP2‐KD (*n* = 24) oocytes. Data in (B), (D), and (E) were expressed as mean ± SEM, and (G) were expressed as mean ± SD of at least three independent experiments. ^***^ *P* < 0.001.

To ask whether the unique role of BFSP2 in mouse oocyte meiosis is conserved across species, we used porcine oocytes as a model. We first observed the subcellular localization of BFSP2 in porcine oocytes. As shown in Figure S7A (Supporting Information), consistent with the results in mouse oocytes, BFSP2 also located to the spindle region in porcine oocytes (Figure S7A, Supporting Information). Immunoblotting analysis confirmed that RNAi‐based gene silencing approach effectively deplete BFSP2 in porcine oocytes (Figure S7B, Supporting Information). We then evidenced that depletion of BFSP2 in porcine oocytes resulted in the reduced PBE rate, elevated spindle/chromosome abnormality rate, and increased MI plate width (**Figure** **7**), as shown in mouse oocytes. These observations validated that the function of BFSP2 during oocyte meiosis is highly conserved across species, at least between mice and pigs.

## 3 Discussion

The IF proteins BFSP1 and BFSP2 are the lens‐specific member of IF superfamily. They form heteropolymers and are highly expressed in differentiated lens fiber cells and lens epithelium.^[^ ^22^, ^47^ ^]^ Therefore, these two proteins are considered as the essential cytoskeletal components of lens fiber and epithelial cells. The absence or mutation of either protein leads to the development of cataracts.^[^ ^18^, ^48^, ^49^, ^50^ ^]^ Previous studies have found that in mouse lens epithelial cells, BFSP1 and BFSP2 co‐localize with actin but do not appear to co‐localize with α‐tubulin.^[^ ^22^ ^]^ Interestingly, we recently reported that BFSP1 drives the asymmetric division of oocytes via regulating spindle length, which is achieved by recruiting heat shock protein 90α (HSP90α) to the spindle structure for stabilization of microtubule‐associated protein 1b (MAP1B).^[^ ^51^ ^]^ In this study, our data discovered that BFSP2 is also expressed throughout the meiotic progression of oocyte maturation, and highly enriched on the spindle apparatus, but exerts a different function during oocyte meiosis.

To decipher the specific role of BFSP2 in oocytes, we performed the loss‐of‐function and rescue experiments to assess the effect of BFSP2 on the key cellular events during meiosis. We revealed that BFSP2 is not critical for oocyte meiotic resumption, but drives the completion of meiosis I. Depletion of BFSP2 impairs the spindle assembly to activate the SAC at MI stage, leading to the oocyte MI arrest. This is consistent with previous studies that disorganized spindle‐induced SAC activation often causes the MI arrest in oocytes.^[^ ^52^, ^53^, ^54^ ^]^ In addition, BFSP2 depletion severely compromises K‐M attachments in oocytes, consequently resulting in the high occurrence of aneuploidy. Hence, these data highlight the requirement of BFSP2 in forming the high‐quality matured oocytes.

The most important finding in our study is that we elucidated the action mechanisms underlying the function of BFSP2 during meiotic spindle assembly. IP/MS analysis identifies a large number of microtubule‐associated proteins as the BFSP2 binding proteins, supporting the conclusion that BFSP2 plays vital roles in the spindle organization. Notably, CLTC exhibits a high affinity for BFSP2. Previous studies have reported that CLTC localizes to the spindle apparatus for stabilizing kinetochore‐associated spindle fibers by acting as an inter‐MT bridge, and promoting chromosome congression in somatic cells.^[^ ^55^, ^56^, ^57^, ^58^, ^59^ ^]^ In particular, CLTC has been found to participate in the meiotic spindle assembly through microtubule‐associated mechanisms during oocyte maturation in several species,^[^ ^36^, ^37^, ^38^ ^]^ which further prompts us to clarify the relationship between BFSP2 and CLTC in oocytes. Additional investigations illustrate that lack of BFSP2 reduces the protein levels of CLTC, and restoration of CLTC levels can compensate for the meiotic defects caused by BFSP2 depletion, attesting that CLTC is the downstream factor mediating the meiotic function of BFSP2 in oocytes.

Another interesting question is how BFSP2 affects CLTC protein stability. In MS data, we took notice of HSC70, a molecular chaperone, that exert a critical function in facilitating protein folding, maintaining structural and functional integrity, and sustaining proteostasis.^[^ ^60^, ^61^, ^62^ ^]^ Previous reports have shown that HSC70 associates with tubulin to take part in the dynamics and function of microtubules.^[^ ^46^, ^63^ ^]^ In line with them, our findings revealed that HSC70 distributes in the spindle region in oocytes, and BFSP2 is responsible for the localization of HSC70 on the spindle apparatus to stabilize CLTC, and thus promoting the meiotic spindle assembly.

In conclusion, we provide a body of evidence to corroborate that IF protein BFSP2 functions as a spindle assembly controller during oocyte meiotic maturation. In normal oocytes, BFSP2 localizes to the spindle apparatus to recruit the molecular chaperone HSC70 for stabilizing CLTC, which in turn regulates the spindle assembly, drives the meiotic progression, and maintains the chromosome euploidy. On the contrary, in BFSP2‐depleted oocytes, HSC70 cannot locate to the spindle apparatus to stabilize CLTC, and thus leads to the defective spindle assembly, meiotic maturation arrest, and aneuploidy (Figure S8, Supporting Information). Our findings unveil a noncanonical role of BFSP2 during oocyte meiosis beyond its conventional function in the lens fiber cells.

## 4 Experimental Section

### Animals

All mouse protocols and experimental procedures (NJAU.No20220927181) were approved by the Animal Research Institute Committee of Nanjing Agricultural University, China. 6‐8‐week‐old ICR female mice were maintained under controlled conditions of temperature (20 to 23 °C) and light (12 h light‐dark cycle) with free access to food and water throughout the study.

### Antibodies

Rabbit polyclonal anti‐BFSP2 antibody (Cat# A13055) was purchased from Abclonal (Wuhan, China); rabbit monoclonal anti‐vimentin antibody (Cat# A19607) was purchased from Abclonal (Wuhan, China); mouse monoclonal anti‐α‐tubulin‐FITC antibody (Cat# F2168) was purchased from Sigma–Aldrich (St. Louis, MO, USA); goat polyclonal anti‐BUBR1 antibody (Cat# ab28193) was purchased from Abcam (Cambridge, MA, USA); human anti‐centromere antibody was (Cat# CA95617) purchased from Antibodies Incorporated (Davis, CA, USA); rabbit polyclonal anti‐CLTC antibody (Cat# 26523‐1‐AP), rabbit polyclonal anti‐HA antibody (Cat# 51064‐2‐AP), mouse monoclonal anti‐β‐Actin antibody (Cat# 66009‐1‐1g), and mouse monoclonal anti‐GAPDH antibody (Cat# 60004‐1‐lg) were purchased from Proteintech (Rosemont, IL, USA).

### Mouse Oocyte Collection and In Citro Culture

As previously described,^[^ ^64^ ^]^ fully‐grown oocytes arrested at GV stage were collected from the ovaries of female mice in M2 medium, and then cultured in M16 medium under liquid paraffin oil at 37 °C in an atmosphere of 5% CO_2_ incubator until they reached the desired developmental stage.

### siRNA Knockdown

As previously described,^[^ ^64^ ^]^ BFSP2‐targeting siRNA oligos (GenePharma, Shanghai, China) were diluted with water to provide a working concentration of 25 µm, and then ≈5 to 10 pl of oligos were microinjected into the cytoplasm of fully grown mouse GV oocytes using a Narishige microinjector. A non‐targeting siRNA oligo was injected as a control. To facilitate the degradation of mRNA by siRNA, mouse oocytes were arrested at GV stage in M16 medium containing 50 µm 3‐Isobutyl‐1‐methylxanthine (IBMX) for 22 h and then transferred to IBMX‐free M16 medium to resume the meiosis for subsequent experiments. The siRNA sequences were listed in Table S1 (Supporting Information).

### mRNA Construct and In Vitro Transcription

BFSP2 cDNA and CLTC cDNA were subcloned into pcDNA3.1/6×HA vector, respectively. As previously described,^[^ ^64^ ^]^ capped mRNA was synthesized from linearized plasmid using T7 High Yield RNA Transcription kit (Vazyme, Nanjing, China; Cat# DD4201), and purified with MEGAclear kit (ThermoFisher Scientific, Waltham, MA, USA; Cat# AM1908). Typically, 10–12 pl of 0.5–1.0 µg µL^−1^ mRNA was injected into oocytes and then arrested at GV stage in M16 medium containing 50 µm IBMX for 4 h, allowing enough time for translation, followed by releasing into IBMX‐free M16 medium for further study.

### Immunofluorescence Staining and Confocal Microscopy

As previously described,^[^ ^65^ ^]^ oocytes were fixed in 4% paraformaldehyde in PBS (pH 7.4) for 30 min and permeabilized in 0.5% Triton‐X‐100 for 20 min at room temperature (RT). Oocytes were then blocked with 1% BSA‐supplemented PBS for 1 h and incubated with BFSP2 (1:100), vimentin (1:100), α‐Tubulin‐FITC (1:500), BUBR1 (1:100), CREST (1:200), CLTC (1:100), HA (1:100), or HSC70 (1:100) antibodies, respectively at 4 °C overnight. After washing in PBST, oocytes were incubated with a corresponding secondary antibody for 1 h at RT, and then counterstained with 10 µg mL^−1^ Hoechst 33342 or propidium iodide (PI) for 10 min. Lastly, oocytes were mounted on glass slides and imaged by laser confocal microscope (LSM 900, Carl Zeiss, Germany). The quantification of fluorescence intensity was performed as described previously.^[^ ^66^ ^]^

### Immunoprecipitation and Immunoblotting

As previously described,^[^ ^64^ ^]^ immunoprecipitation was carried out using 20 mouse ovaries according to the instruction for ProFound Mammalian CoImmunoprecipitation Kit (ThermoFisher Scientific). For immunoblotting, 150‐200 oocytes were lysed in 4 × LDS sample buffer (ThermoFisher Scientific) containing protease inhibitor, and then separated on 10% or 4%–12% Bis‐Tris precast gels and transferred onto PVDF membranes. The blots were blocked in TBST containing 5% low fat dry milk for 1 h at RT and then incubated with BFSP2 (1:1000), CLTC (1:1000), HA (1:5000), HSC70 (1:2000), β‐Actin (1:5000), or GAPDH (1:5000) antibodies, respectively at 4 °C overnight. After three times of wash in TBST, the blots were incubated with HRP (horse radish peroxidase) conjugated secondary antibodies for 1 h at RT. Chemiluminescence signals were detected with ECL Plus (ThermoFisher Scientific) and protein bands were acquired by Tanon‐3900 Chemiluminescence Imaging System (Tanon, Beijing, China). Band intensities were quantified using ImageJ software and normalized to loading controls.

### Chromosome Spreading

As previously described,^[^ ^65^ ^]^ oocytes were incubated in Tyrode's buffer (pH 2.5) for ≈30 s at 37 °C to remove zona pellucidae. After recovery in M2 medium for 10 min, oocytes were fixed in a drop of 1% paraformaldehyde with 0.15% Triton X‐100 on a glass slide. After air drying, chromosomes were counterstained with PI and examined by confocal microscopy.

### Liquid Chromatography‐MS/MS and Data Analysis

As previously described,^[^ ^51^ ^]^ the iTRAQ technology was carried out with the help of Gene Create Biolabs Inc (Wuhan, China). For this purpose, total proteins were isolated from each biological sample. The protein concentration was measured using the Bradford method. Following digestion with Trypsin Gold (Promega, Madison, WI, USA), the peptides were dried, reconstituted in 0.5 m triethylammonium bicarbonate (TEAB) buffer (ThermoFisher Scientific), and then processed with 8‐plex iTRAQ reagent (ThermoFisher Scientific), according to the manufacturer's protocol.

The mixed peptides were fractionated using the Ultimate 3000 HPLC system (Thermo DINOEX, USA). Mass spectrometry data were generated using the TripleTOF 5600 + liquid mass spectrometry system (SCIEX, USA) coupled with the Eksigent nanoLC system (SCIEX). TripleTOF 5600plus liquid chromatography and mass spectrometry system (SCIEX) was used for mass spectrometry data acquisition.

The original MS/MS file data were analyzed by ProteinPilot Software v4.5, with the unused score ≥ 1.3 (corresponding to proteins identified with ≥ 95% confidence). An automatic decoy database search strategy was employed to estimate the false discovery rate (FDR) using the PSPEP (Proteomics System Performance Evaluation Pipeline Software, integrated with the ProteinPilot Software). Fold changes (FCs) were calculated as the average comparison pairs among biological replicates to determine differentially expressed proteins (DEPs). Proteins with an FC greater than 2 (upregulate ≥ 2.00 and down‐regulate ≤ 0.50) and a Q‐value less than 0.05 were considered significantly differentially expressed.

### Molecular Modeling

3D predictive structures of mouse BFSP2 (Q6NVD9) and CLTC (Q68FD5) were extracted from the AlphaFold databank. As previously described,^[^ ^51^ ^]^ protein‐protein docking was performed by HDOCK server, and docking model was displayed using pymol. To determine the reliability of the docking model, two factors need to be considered, including docking score and the confidence score. The docking score reflects the binding affinity between the ligand and the receptor. A lower (more negative) score typically suggests a stronger binding interaction. The confidence score in docking refers to a numerical value that indicates the reliability or accuracy of a docking prediction. When the confidence score is above 0.7, the likelihood of binding is high, and when the confidence score is below 0.5, the likelihood of binding is considered low.

### VER‐155008 Treatment

VER‐155008 powder (MedChemExpress, Monmouth Junction, NJ, USA; HY‐10941) was dissolved in dimethyl sulfoxide (DMSO) to prepare a 5.2 mm stock solution, and then diluted to a working concentration of 2.6 µm using M16 medium. The oocytes were incubated in M16 medium containing VER‐155008 for 24 h. The concentration of DMSO in the culture medium was less than 0.1%.

### Porcine Oocyte Collection and In Vitro Maturation

As previously described,^[^ ^65^ ^]^ abattoir‐derived porcine ovaries were transported to the laboratory within 2 h in a physiological saline containing penicillin G/streptomycin sulphate. Cumulus‐oocyte complexes (COCs) were isolated from the follicles (3–6 mm in diameter) using a disposable syringe with a 20‐gauge needle. Oocytes with a compact cumulus cells were used for in vitro maturation (IVM) in TCM‐199 (ThermoFisher Scientific) supplemented with 10 ng mL^−1^ EGF, 5 µg mL^−1^ insulin, 0.2 mm pyruvate, 0.6 mm cysteine, 10% porcine follicular fluid, 10 IU mL^−1^ of each eCG and hCG, and 25 µg mL^−1^ kanamycin. 20‐30 GV oocytes were cultured for 28 h to MI stage and 44‐48 h to MII stage in 100 µL TCM‐199 covered with mineral oil at 38.5 °C, 5% CO_2_.

### Statistical Analysis

All statistical data from at least three independent experiments were presented as mean ± SEM or SD unless otherwise stated, and the number of samples used in each group was labeled in parentheses as (n). Data were analyzed by two‐tailed unpaired *t*‐test, which is provided by GraphPad Prism 10 statistical software. *P* < 0.05 was considered as statistical significance.

## Conflict of Interest

The authors declare no conflict of interest.

## Author Contributions

B.X. conceived and designed the research; Y.L., Z.Z., and Y.Z. performed the experiments; Y.L. and B.X. analyzed the data; Y.L. and B.X. wrote the manuscript.

## Supporting information



Supporting Information

Supporting Information

## Data Availability

The data that support the findings of this study are available in the supplementary material of this article.
