# Supplementary material for: Intermediate Filament Protein BFSP2 Controls Spindle Formation via HSC70‐Mediated Stabilization of CLTC During Oocyte meiosis
Source: Adv Sci (Weinh). 2025 Jul 2;12(37):e06639. doi: 10.1002/advs.202506639 (PMC12499473; doi:10.1002/advs.202506639)
Supplement: Supplementary file 1 — Supporting Information [file ADVS-12-e06639-s002.doc]

**Supplementary Information**

**Intermediate filament protein BFSP2 controls spindle formation via HSC70-mediated stabilization of CLTC during oocyte meiosis**

Yu Li, Zihao Zhang, Yu Zhang, Bo Xiong


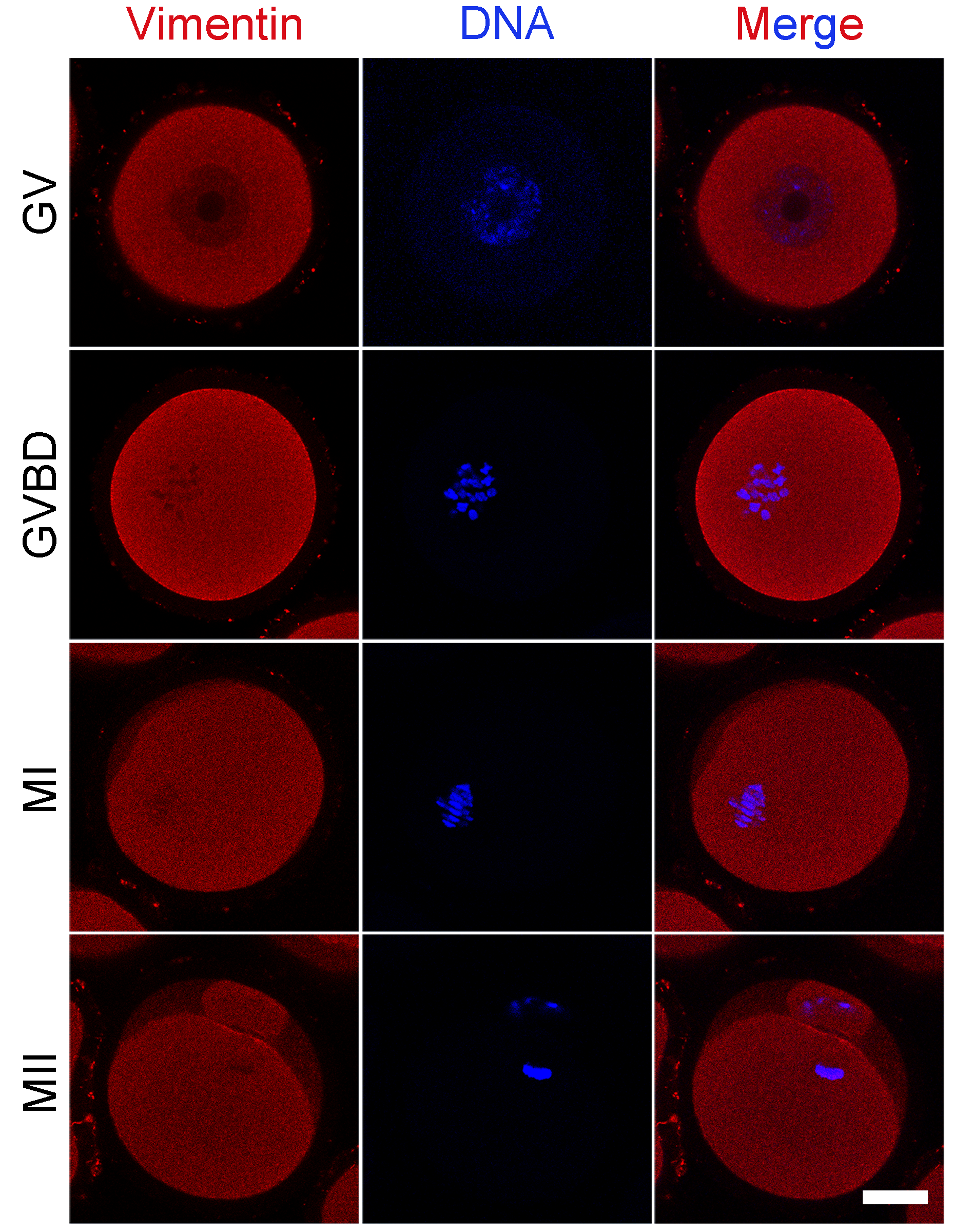


**Figure S1. Localization of Vimentin in mouse oocytes.** Fluorescence images of Vimentin localization in oocytes. Mouse oocytes at GV, GVBD, MI and MII stages were immunostained with Vimentin antibody and counterstained with Hoechst. Scale bar, 20 μm.


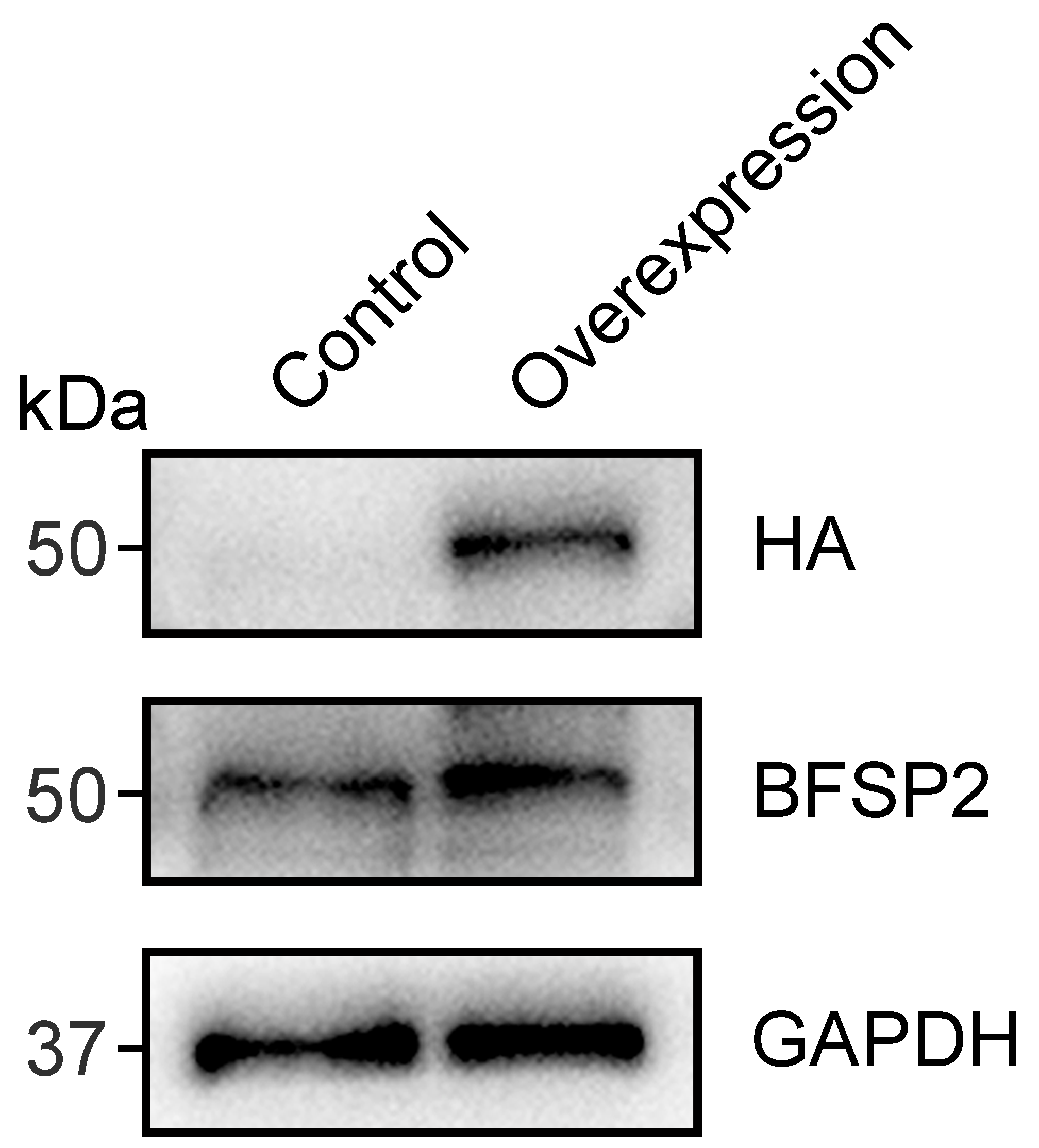


**Figure S2. Expression of BFSP2-6×HA in mouse oocytes.** Immunoblotting analysis of BFSP2 and HA tag in control and BFSP2-6×HA-expressed oocytes. The blots were probed with BFSP2, HA and GAPDH antibodies, respectively.


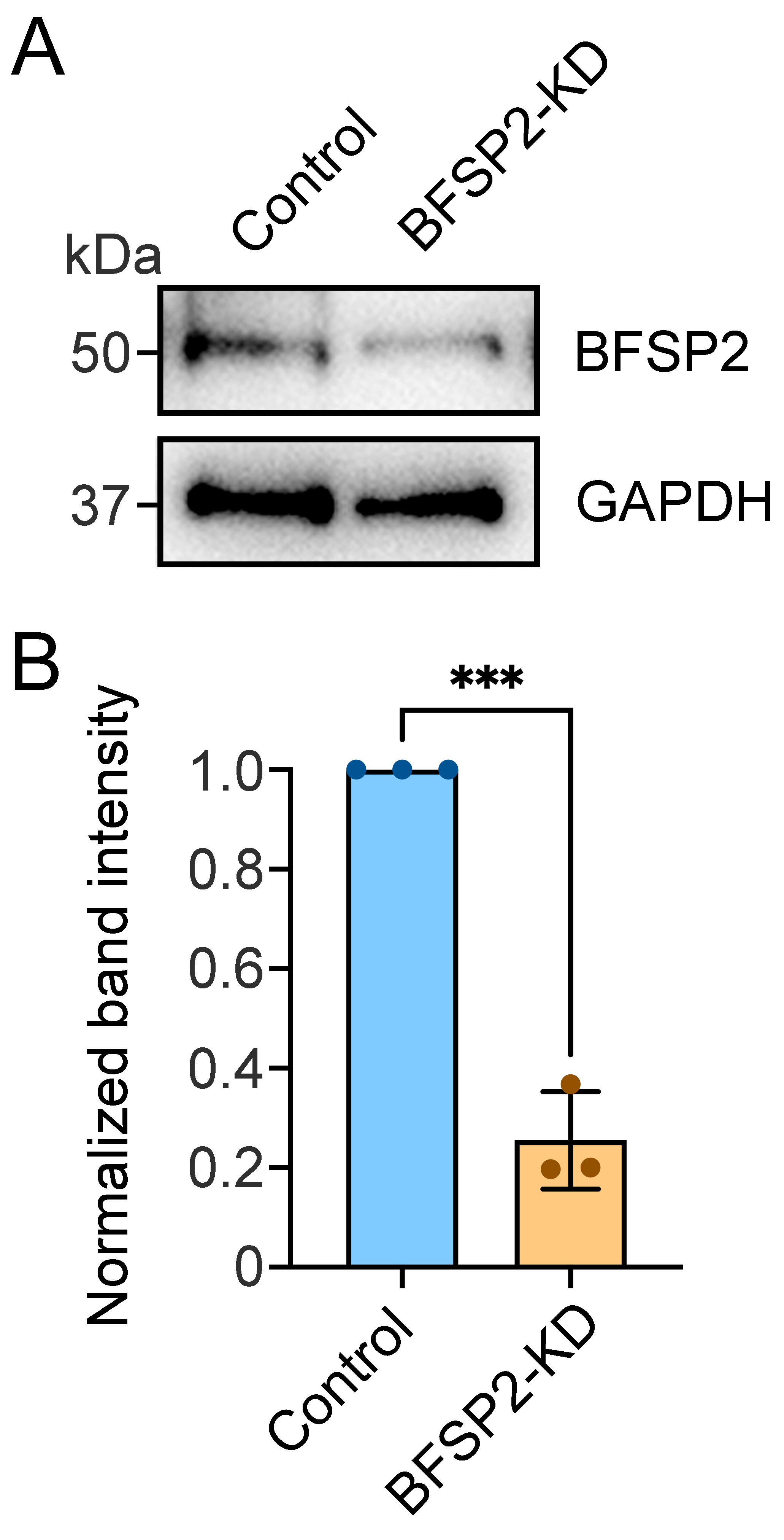


**Figure S3. Knockdown efficiency of BFSP2 in mouse oocytes.** (A) Immunoblotting analysis of BFSP2 protein levels in control and BFSP2-KD oocytes. The blots were probed with BFSP2 and GAPDH antibodies, respectively. (B) Quantification of BFSP2 protein levels in control and BFSP2-KD oocytes. The band intensity of BFSP2 was normalized with that of GAPDH. Data in (B) were expressed as mean ± SD of at least three independent experiments. ***P < 0.001.


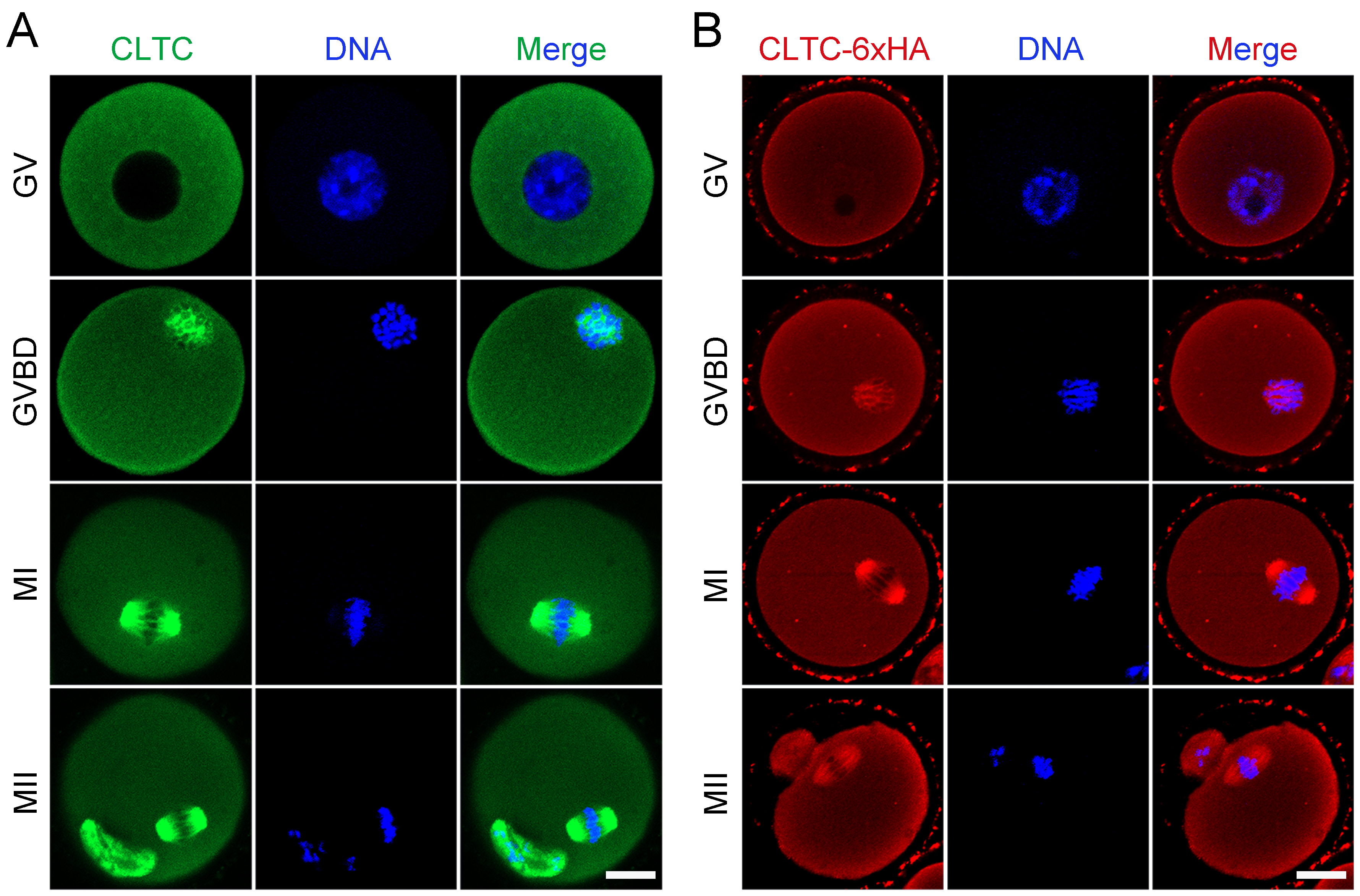


**Figure S4. Localization of endogenous and exogenous CLTC in mouse oocytes.** (A) Fluorescence images of CLTC localization in oocytes. Mouse oocytes at GV, GVBD, MI, and MII stages were immunostained with CLTC antibody and counterstained with Hoechst. Scale bar, 20 μm. (B) Fluorescence images of CLTC-6×HA localization in oocytes. Mouse oocytes at GV, GVBD, MI, and MII stages were immunostained with HA antibody and counterstained with Hoechst. Scale bar, 20 μm.


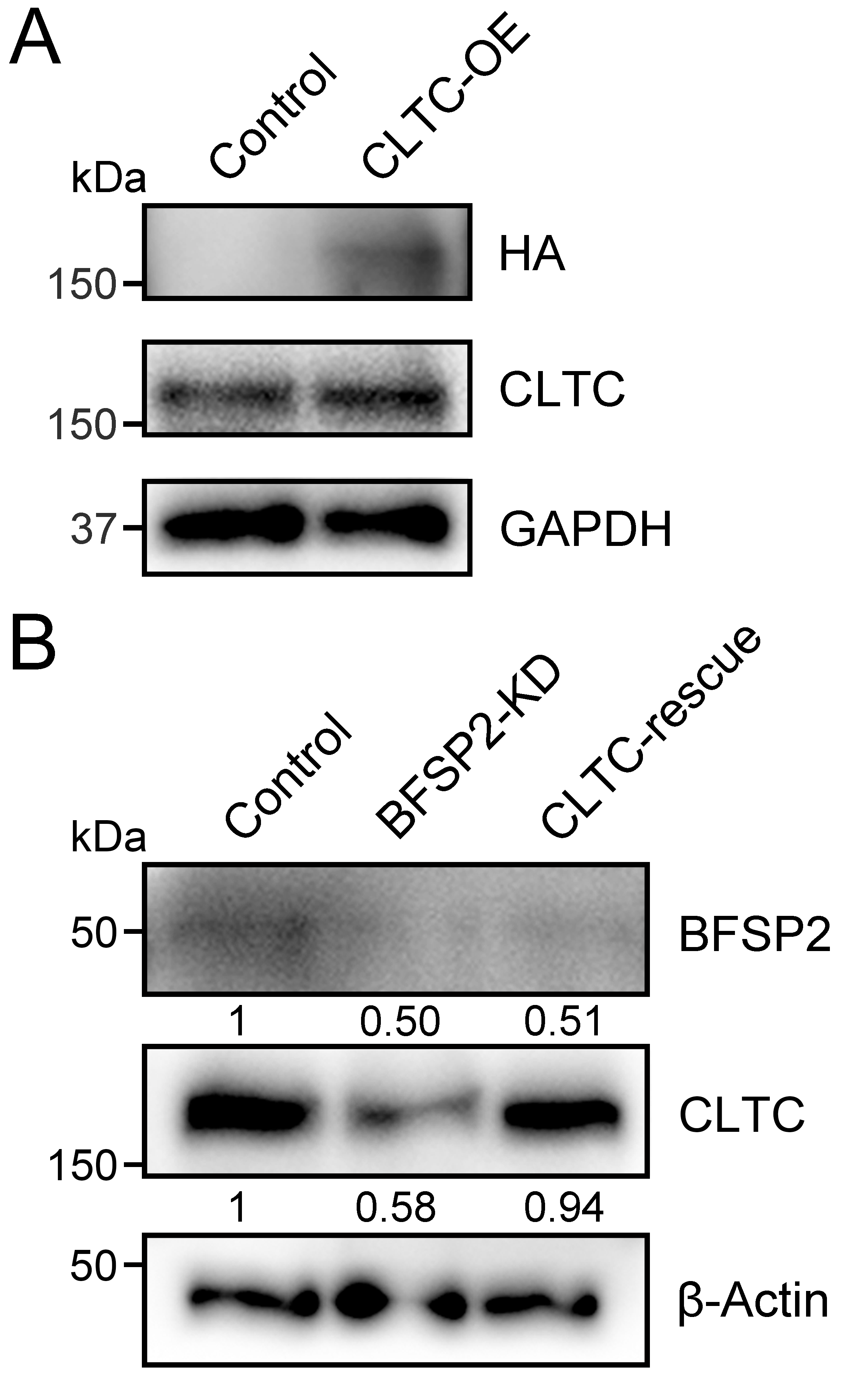


**Figure S5. Expression of CLTC-6×HA in control and BFSP2-KD oocytes.** (A) Immunoblotting analysis of CLTC and HA tag in control and CLTC-6×HA-expressed oocytes. The blots were probed with CLTC, HA and GAPDH antibodies, respectively. (B) Immunoblotting analysis of CLTC protein levels in control, BFSP2-KD, and CLTC-rescued (BFSP2-KD + CLTC-6×HA) oocytes. The blots were probed with BFSP2, CLTC, and β-Actin antibodies, respectively.


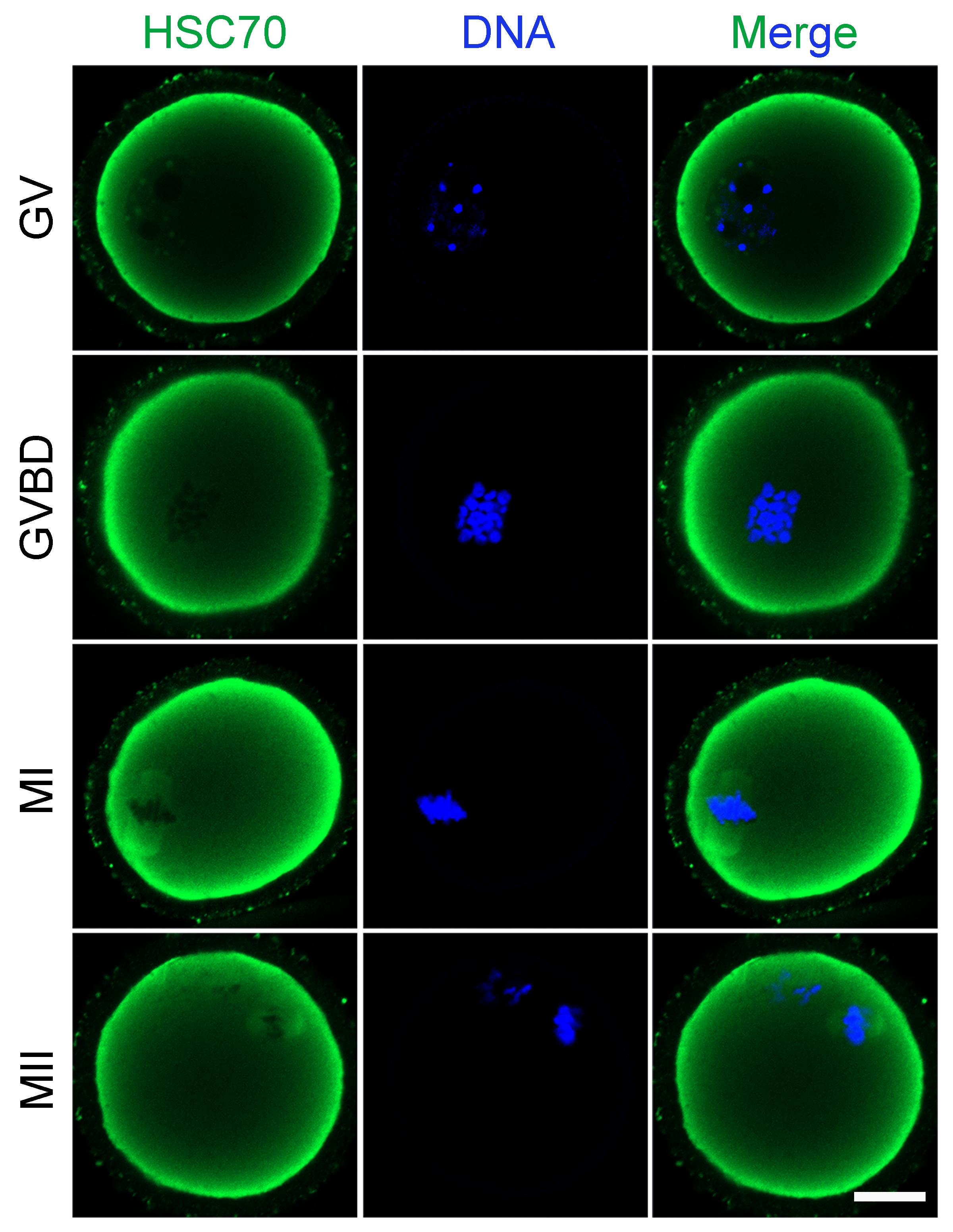


**Figure S6. Localization of HSC70 in mouse oocytes.** Fluorescence images of HSC70 localization in oocytes. Mouse oocytes at GV, GVBD, MI and MII stages were immunostained with HSC70 antibody and counterstained with Hoechst. Scale bar, 20 μm.


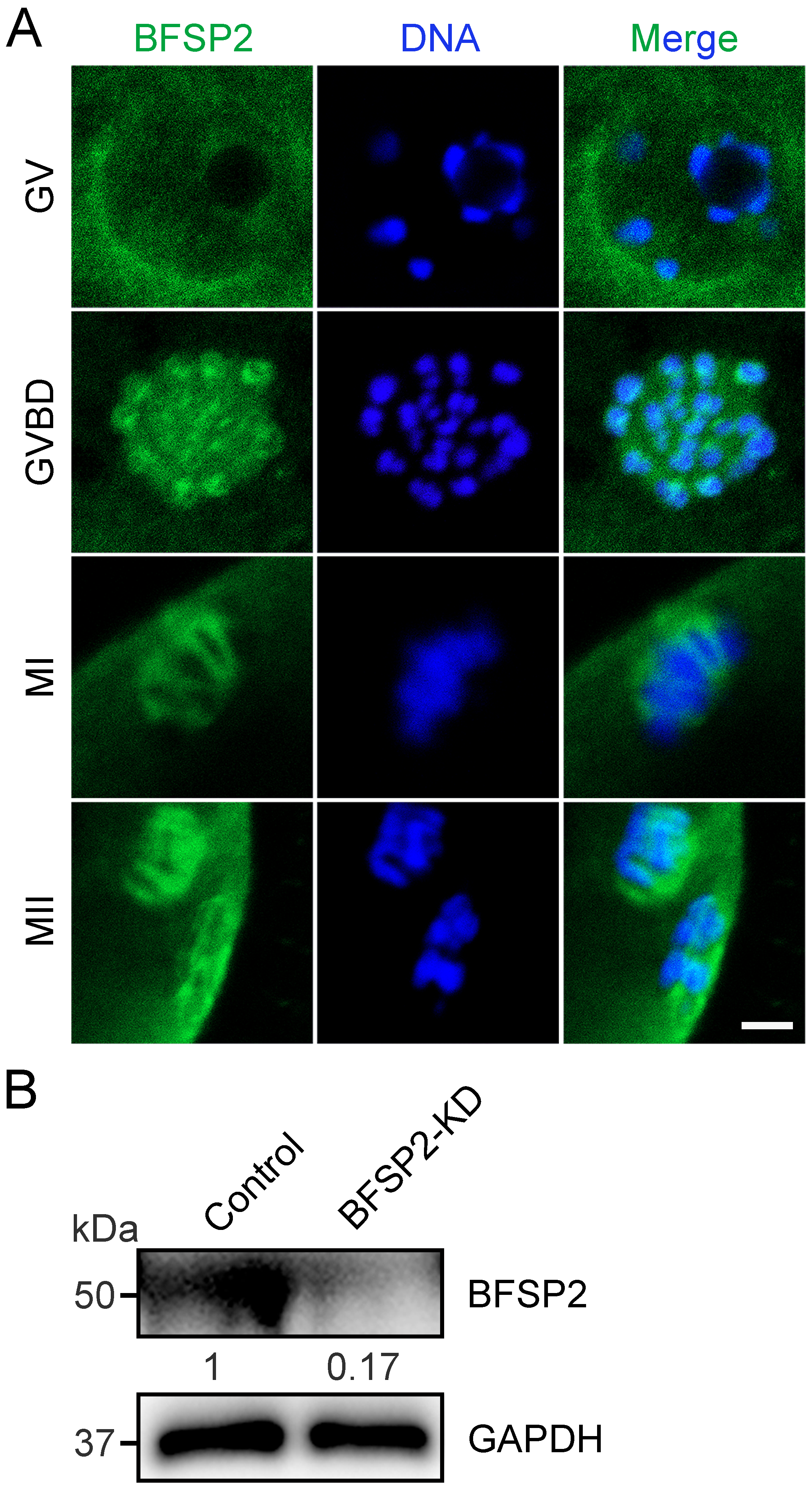


**Figure S7. Localization and expression of BFSP2 in porcine oocytes.** (A) Fluorescence images of BFSP2 localization in porcine oocytes. Porcine oocytes at GV, GVBD, MI and MII stages were immunostained with BFSP2 antibody and counterstained with Hoechst. Scale bar, 5 μm. (B) Immunoblotting analysis of BFSP2 protein levels in control and BFSP2-KD porcine oocytes. The blots were probed with BFSP2 and GAPDH antibodies, respectively.

**
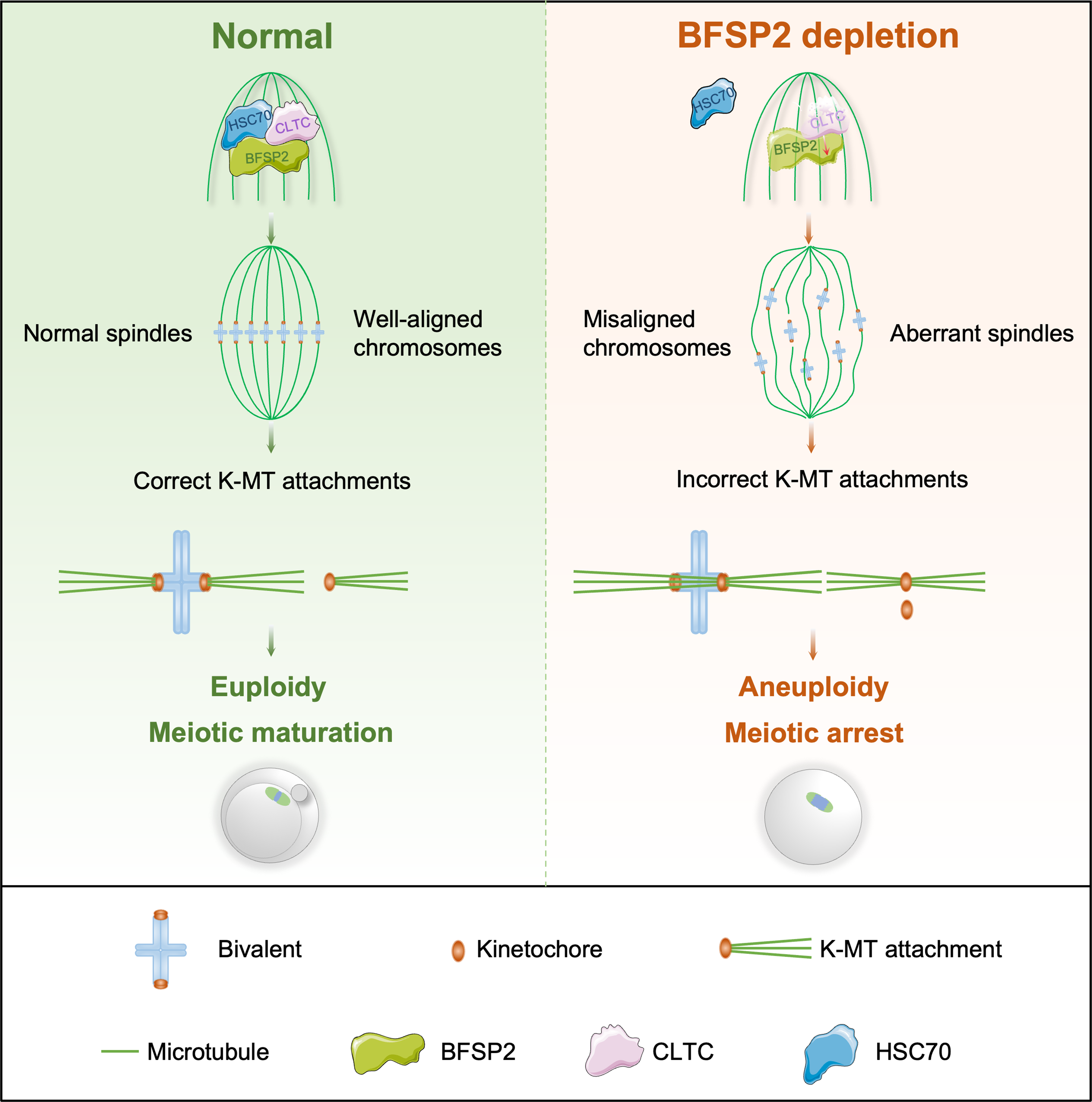
**

**Figure S8. Working model for the action of BFSP2 during meiotic spindle assembly in oocytes.**

**Table S1. Primer sequences of genes for siRNA oligos**

| **Gene** | **sense (5’-3’)** | **antisense (5’-3’)** |
| --- | --- | --- |
| *Bfsp2-1* (Mus) | GCUGCCUAGUGGAAUAUAUTT | AUAUAUUCCACUAGGCAGCTT |
| *Bfsp2-2* (Mus) | CGGAUCUGGAGCAUCAAAUTT | AUUUGAUGCUCCAGAUCCGTT |
| *Bfsp2-3* (Mus) | CUCCAAGCUAAGCAACAGATT | UCUGUUGCUUAGCUUGGAGTT |
| Control (Mus) | UUCUCCGAACGUGUCACGUTT | ACGUGACACGUUCGGAGAATT |
| *BFSP2* (Sus) | GACGACAUCCUUGAGACCATT | UGGUCUCAAGGAUGUCGUCTT |
| Control (Sus) | UUCUCCGAACGUGUCACGUTT | ACGUGACACGUUCGGAGAATT |
